# Different doses of recombinant FSH and determining parameters of oxidative stress

**DOI:** 10.5937/jomb0-46156

**Published:** 2024-04-23

**Authors:** Lidija Tulić, Ivan Tulić, Jelena Stojnić, Jovan Bila, Željka Vuković, Boba Kotlica

**Affiliations:** 1 University of Belgrade, School of Medicine, Clinical Center of Serbia, Clinic for Gynecology and Obstetrics, Department of In Vitro Fertilization, Belgrade; 2 Clinical Center of Serbia, Clinic for Gynecology and Obstetrics, Belgrade; 3 University of Belgrade, School of Medicine, Belgrade

**Keywords:** in vitro fertilization, oxidative stress, gonado tropin dose, in vitro oplo|enje, oksidativni stres, doza gonadotropina

## Abstract

**Background:**

This study aimed to examine if there is a connection between recombinant FSH dose and OS parameters in serum and the impact on IVF outcome.

**Methods:**

This study consisted of 101 participants that went through IVF procedures. Parameter that were tested are SOD, SH groups and MDA. Serum samples were drawn before stimulation and on the last day of ovarian stimulation.

**Results:**

Two groups were formed according to the dose of gonadotropins (rFSH). In both groups there were no significant differences in live-birth rate and miscarriage. In both groups mean serum MDA and SH-groups were significantly higher after ovarian stimulation, but mean serum SOD was significantly lower when compared to values before stimulation. There were less patients without OS before stimulation.

**Conclusions:**

Our results suggest that there is a difference in serum concentration in groups SOD, SH groups and MDA at the beginning and at the end ovarian stimulation. On the other hand, dose of rFSH is not related with change of parameters for oxidative stress, quality of oocytes, embryos, fertilization, pregnancies, and miscarriage rate. Patients without oxidative stress before the IVF procedure needed lower doses of gonadotropins during stimulation.

## Abstract (sr)

**Uvod:**

Cilj rada je bio istraživanje povezanost doze rekombinantnog FSH sa parametarima oksidativnog stresa, kao i uticaj na ishod IVF postupka.

**Metode:**

U studiju je uključena 101 pacijentkinja koja je lečena IVF postupkom. Parametri oksidativnog stresa koji su iza brani su: SOD, SH grupe i MDA. Uzimani su uzorci krvi/seruma pre početka i poslednjeg dana stimulacije jajnika.

**Rezultati:**

Zavisno od doze gonadotropina (rFSH) koju su primile, ispitanice su podeljene u dve grupe. U obe grupe nije bilo značajnih razlika u stopi živorođenosti i pobačaja. Srednji serumski MDA i SH grupe bili su značajno viši, a srednji serumski SOD bio je značajno niži nakon stimulacije u poređenju sa vrednostima pre stimulacije jajnika i to u obe grupe. Bilo je manje žena bez OS pre stimulacije.

**Zaključak:**

Postoji razlika u koncentracijama SOD, SH grupa i MDA u uzorcima na početku ciklusa u odnosu na one uzete na kraju stimulacije jajnika. Doza rFSH nije povezana sa promenom parametara OS, sa procenjenim kvalitetom jajnih ćelija i embriona, niti oplodnjom, trudnoćama i stopom pobačaja. Pacijentkinjama bez oksidativnog stresa pre ulaska u IVF postupak bile su potrebne niže doze gonadotropina tokom stimulacije.

## Introduction

The number of couples with infertility problems is continuously increasing. It is estimated that 20 percent of couples in Europe have a problem with getting pregnant [1]. There are different causes of infertility: female factor, male factor, unexplained cause of infertility, etc. One of the underlying factors could be oxidative stress. Oxidative stress (OS) happens because of the imbalance between pro-oxidants and antioxidants. The problem occurs when there is an increase in the concentration of reactive oxygen species (ROS) and/or decrease of antioxidant defense [2] [3]. Fluids that are influenced by oxidative stress are: tubal, follicular and peritoneal [4] [5]. Also it has impact on oocytes, embryos [6] [7] [8] and sperm [9] [10] which may be the possible reason of infertility. It is still not clear if OS disturbs endometrial receptivity [11]. Several studies have linked endometriosis, polycystic ovary syndrome, spontaneous miscarriages, preeclampsia, embryopathies, preterm delivery, intrauterine growth restriction of the fetus, and idiopathic infertility with OS [12] [13] [14].

In vitro fertilization (IVF) is widely used as a treatment of infertility. The fact is that the outcome of IVF procedure is multifactorial and it is influenced by certain factors like: age, ovarian reserve, body mass index, reasons of infertility, stimulation protocols, oocyte number, quality of oocytes and embryos, morphological characteristics of sperm and its number. One of the possible causes that may have an influence on the IVF outcome is oxidative stress. In IVF there may be multiple sources of ROS, including gametes [15], the time of insemination [16] [17], assisted reproductive techniques (ART) that are used, pH and osmotic shock, light, temperature changes, media, supplements, etc.

Conventional ovarian stimulation protocols with gonadotropins (GT) that precede IVF procedure are used to achieve maximum oocyte yields. Recombinant follicle-stimulating hormone (rFSH) which had 99% purity is much the same as FSH that human body produces [18]. It has been suggested that a smaller total dose and fewer days of stimulation are needed with rFSH contrasted to highly purified urinary-derived gonadotrophins [19]. Anyways, ovarian stimulation represents stress in some sense. GT stimulation can directly affect the markers of OS [20].

This study aimed to examine the link between the dose of recombinant follicle stimulating hormone (rFSH), superoxide dismutase (SOD), sulfhydryl groups (SH) groups and malondialdehyde (MDA) in serum, and the influence of lower and higher doses of rFSH on IVF outcome.

## Materials and methods

### Study subjects

Our study consisted of 101 women that went through IVF procedures at the Clinic for Gynecology and Obstetrics, Clinical Center of Serbia. All subjects allowed to be involved in the study which they confirmed by signing an informed consent. The Ethics Committee of the School of Medicine, University of Belgrade approved the study. Patient selection method and clinical protocols are already elaborated in detail [21]. In short, the criteria for including the patient in the study were: 18–40 years of age, BMI 18–30 kg/m^2^ and regular menstrual cycles. Patients did not have endometriosis stage III and Ivor any other medical illness. All infertility factors and demographic characteristics like patients' age, BMI, infertility duration and smoking status were determined. The protocols that were applied are: short GnRH antagonist with contraceptives pretreatment, or without it or long GnRH agonist. In all protocols rFSH - folitropin (Gonal-F, Serono, Switzerland) was used for stimulation. Patients on a long agonist protocol used Triptoreline (Diphereline, Ipsen Pharma Biotech, France) from the mid-luteal phase of previous cycle. After suppression of pituitary, rFSH stimulation was started (150–300 IU/day). Patients on a short antagonist protocol started stimulation with rFSH on second or third day of the cycle, after normal ultrasound findings and normal basal hormone level (FSH, luteinizing hormone (LH), progesterone (P4), estradiol (E2), and anti-Mullerian hormone (AMH)). Cetrorelix (Cetrotide, Merck Serono, Germany) was injected when the leading follicle achieved a diameter of 14 mm. We did serial ultrasounds and estradiol measurements until two or more follicles reached size > 18 mm, at which point recombinant human chorionic gonadotropin (rhCG), (Ovitrelle, Merck, The Netherlands) was given at a dose of 250 micrograms. Retrieval of the oocyte was done 34–36 hours after rhCG administration. For insemination IVF, ICSI or a combined method was used. We performed embryo transfer on day two or day three. In order to support luteal phase, progesterone was given until the 12th week of gestation. Beta hCG was done 2 weeks after the embryo transfer. Pregnancies were verified at the 6th week of gestation by ultrasound.

### Sample collection

Blood samples were taken from patients and were centrifuged corresponding to the manufacturer’s instruction. First sample was taken at the begging of the cycle (day 2–4), while second sample was taken on the day of administration of the hCG. After the preparation of serum samples, basal hormonal status was determined: FSH, LH, P4, E2, and AMH as well as OS parameters: the activity of total superoxide dismutase (SOD), the concentration of total sulfhydryl (SH) groups and malondialdehyde (MDA). FSH, LH, E2 and P4 were analyzed by chemiluminescent immunoassay, but AMH was measured by enzymelinked immunosorbent assay (ELISA).

The freezing temperature of plasma/serum was -70°C, homogenization and centrifugation weredone as a preparation for the analysis. Bradford method was used to determine proteins, which uses colors binding method [22]. The analysis of MDA was based on the reaction of MDA with thiobarbituric acid in acidic medium for 15 minutes at 95°C in a water bath. Method of Misra and Fridovich was used to analyse the activity of SOD in alkaline [23]. Ellman's method was used to determine total concentration of SH groups [24].

### Statistical analysis

Variables with normal distribution were given as standard deviation and arithmetic mean while others were represented as median and interquartile range. Kolmogorov-Smirnov analysis was used to test relative and absolute frequency for categorical variables and the distribution. Student t-test was used to compare the mean values of independent groups of data, while for differences between subgroups ANOVA analysis with Tukey's post hoc test was utilized. The Mann Whitney and Kruskal-Wallis with post hoc tests were implemented to see if there is a statistically relevant significance between groups. In order to test data without normal distribution Wilcoxon signed-rank test was used and Chi-square test for analysis of categorical values. Statistical Package for the Social Sciences (SPSS 22) was implemented and for all tests statistically significant difference was considered for values less than 0.05.

## Results

The patient's characteristics, levels of basal hormones, and data of IVF outcome are given in Table 1.

**Table 1:** Patients characteristics, basal hormonal status and IVF outcome.

Parameters	Mean value
Age, years	34.6 ± 3.7
BMI, kg/m^2^	22.16 ± 2.82
Smokers, %	27.4
Infertility, years	5 (2–12)
FSH, mIU/mL	7.39 ± 2.5
LH, mIU/mL	5.21 (3.4–6.6)
E2, pg/mL	40.06 (3.00–60.00)
P4 ng/mL	1.53 (0.20–6.40)
AMH, ng/mL	1.43 (0.68–3.12)
Gonadotropin dose, IU	2250.2 ± 604.2
Oocyte number	5.5 (3–11)
Mature oocyte number	4.5 (2–8)
Fertilized oocytes number	3 (1–5)
Fertilization rate, %	59.5 (30.07–786)
Pregnancy rate, %	46
Delivery rate, %	36

On the average the patients were 35 years old, BMI was 22, 27% were smokers, and the duration of infertility at the time of the examination was 5 years. All hormones (FSH, LH, E2, P4) in basal hormonal status were within normal values.

Concerning the other important data, the most frequent infertility cause was male (31%). There were also 22% of tubal infertility, 22% of ovarian, 20% of the unknown and only 5% of combined causes of infertility. The quality of embryos was divided into class A (47.1%), class B (30.6%), class C (9.4%), and A/B class (12.9%).

Patients were divided into two groups based on the FSH dose that they got. Subjects with gonadotropin dose below 75th percentile (<2625 mlU/mL) were in group I (70 patients), while those with gonadotropin dose above 75-th percentile (≥2625 mlU/mL) were in group II (31 patients). In Table 2 the clinical data and demographic features for both groups were presented.

**Table 2:** Demographic features, basal hormonal status and IVF outcome in both groups. Arithmetic mean values ± SD or median (inter-quartile range) a Student t-test; bMann Whitneu U test.

Parameters	rFSH dose		
	Group I (<2625 mlU/mL)	Group II (≥2625 mlU/mL)	P
Age	34.8 ± 4.0	34.4 ± 3.7	0.664^a^
BMI, kg/m^2^	22.01 ± 2.95	22.31 ± 2.69	0.650^a^
Smokers, %	25.4	29.4	0.668
Infertility, years	5 (3–6)	5 (4–7)	0.567^b^
FSH, mIU/mL	7.30 ± 2.49	7.48 ± 2.56	0.750^a^
AMH, ng/mL	1.86 (0.77–3.13)	1.00 (0.41–2.20)	0.021^b^
Oocyte number	6 (3–11)	5 (2–9)	0.311^b^
Mature oocyte number (MII)	5 (2–8)	4 (2–9.75)	0.170^b^
Fertilized oocytes number	3 (1–6)	3 (2–5)	0.695^b^
Fertilization rate %	50.0 (32.0–72.4)	63.6 (36.65–100)	0.689^b^

In group I, values of AMH were significantly higher comparing to group II (1.86 vs. 1.00 ng/mL; p<0.05). The number of fertilized oocytes, fertilization rate, mean total and mature oocytes number were similar in both groups (Table 2). Also, the quality of embryos between groups did not differ. For both groups there were no statistically significant differences in the live–birth rate comparing to the outcome of IVF. The rate of biochemical pregnancies and miscarriage rate for both groups were similar. In group I, after ovarian stimulation mean serum MDA (1.41 vs 1.74 μmol/L, p<0.001) and SH groups were significantly higher (0.24 vs 0.46 mmol/L, p<0.001), while mean serum SOD was significantly lower (17.46 vs. 14.24 U/L, p<0.001) (Table 3). In group II, after ovarian stimulation similar results were observed, mean serum MDA (1.40 vs 1.69 μmol/L, p<0.001) and SH groups (0.23 vs 0.53 mmol/L, p<0.001) were significantly higher, while mean serum SOD was significantly lower (17.81 vs. 14.59 U/L, p<0.001) (Table 4).

**Table 3:** Concentrations of OS parameters at the beginning of the cycle and at the end of stimulation in group I. The median (25th and 75th percentile) are shown. Wilcoxon signed-rank test was used.

Group I (<2625 mlU/mL)	SOD, U/L	SH groups, mmol/L	MDA, μmol/L	P
Cycle day 2–4	17.56(15.60–19.68)	0.24 (0.18–0.28)	1.41 (1.26–1.50)	<0.001
Day of hCG	14.24(12.98–15.63)	0.46 (0.35–0.54)	1.74 (1.26–1.50)	

**Table 4:** Concentrations of OS parameters in the beginning of the cycle and at the end of stimulation in group II. The median (25th and 75th percentile) are shown. Wilcoxon signed-rank test was used.

Group II (≥2625 mlU/mL)	SOD, U/L	SH groups, mmol/L	MDA, μmol/L	P
Cycle day 2–4	17.81 (16.9–19.00)	0.23 (0.21–0.30)	1.40 (1.33–1.53)	<0.001
Day of hCG	14.59 (12.97–15.68)	0.53 (0.42–0.59)	1.69 (1.59–1.84)	

No statistically significant differences were found between two groups in concentrations of SOD, SH groups and MDA after ovarian stimulation (Table 5).

**Table 5:** Concentrations of OS parameters after stimulation between both groups. The median (25th and 75th percentile) are shown. Comparison values after stimulation was performed by Mann Whitney test.

Parameters	rFSH dose		
	Group I (<2625 mlU/mL)	Group II (≥2625 mlU/mL)	P
SOD, U/L	14.24 (12.98–15.63)	14.59 (12.97–15.68)	0.866
MDA, μmol/L	1.74 (1.26–1.50)	1.69 (1.59–1.84)	0.600
SH-groups, mmol/L	0.46 (0.35–0.54)	0.53 (0.42–0.59)	0.061

The group with oxidative stress included all women who before stimulation MDA and SH group concentrations above the 75th percentile and had SOD activity below the 25th percentile. The other women were classified in the group without oxidative stress. Table 6 presents the limit values of examined OS parameters. In the group without OS, there were 46.6% women, which is significantly less than the women with OS before stimulation (53.4%), (p <0.023; test c2). Table 6 shows demographic characteristics, basal hormonal status, number of fertilized oocytes and fertilization rate, number and quality of oocytes in women with and without OS before stimulation. A statistically significant difference in rFSH dose was found between the two study groups (p=0.021). The values are significantly lower in the group without OS comparing to group with OS. Embryo quality was assessed in subjects with and without OS, but there were not significant difference between them. Regardless of current optimal values of oxidative stress parameters, A-grade embryos predominated. Pregnancy was recorded in 47.1% of women without OS versus 42.9% of women with OS. Both groups had similar birth rate.

**Table 6:** Demographic characteristics, basal hormonal status, No and quality of oocytes, No of fertilized oocytes and fertilization rate in women with and without OS. Arithmetic mean values ± SD or median a. Student t-test; bMann Whitneu U test.

Parameters	Oxidative stress before stimulation		
	with OS	without OS	P
Age, years	34.2 ± 3.5	35.1 ± 4.2	0.244^a^
BMI, kg/m^2^	21.43 ± 2.45	22.34 ± 2.92	0.109^a^
Smokers, %	17.9	33.9	0.082
Infertility, years	4 (3–6)	5 (4–7)	0.106^b^
FSH, mIU/mL	7.20 ± 2.82	7.374 ± 2.24	0.734^a^
AMH, ng/Ml	1.54 (0.76–3.43)	1.40 (0.53–3.03)	0.652^b^
rFSH dose, IU	2079.78±575.38	2373.31 ± 597.01	0.021^a^
Oocyte number	6 (3–12)	5 (2–11)	0,504^b^
Mature oocyte number	3.5 (2.3-7.0)	5.0 (2.0–10.0)	0.339^b^
Fertilized oocytes	3 (1–5)	3 (1–5)	0.704^b^
Fertilization rate, %	50.0 (39.8–86.3)	50.0 (25.6–75.5)	0.457^b^

## Discussion

Different protocols of ovarian stimulation and even the stimulation itself in assisted reproduction can lead to disruption of oxidant-antioxidant balance, which lowers the protection of serum from oxidation. Studies about OS determined OS markers in serum and/or follicular fluid (FF). Concentrations of antioxidants in FF were in correlation with the corresponding concentrations in plasma [25]. In other studies a positive correlation [26] [27] was found, indicating that systemic oxidative profile can affect follicular oxidative status.

During ovarian stimulation increased doses of gonadotropins were applied to stimulate multiple follicular developments in one cycle. This obtains a larger number of oocytes and embryos for fresh embryo transfers, cryopreservation, and future embryo transfer [28].

Different studies examined the correlation of pregnancy rate with the number of oocytes [29] [30], the influence of E2 concentrations on IVF outcome [31], the deleterious effect of exogenous gonadotropins development of an embryo. One study analyzed the quality of embryos in natural and stimulated cycles with long protocol and found no difference in rates of fertilization, the number, and degree of fragmentation in blastomeres [32]. In addition, excessive response to ovarian stimulation had no negative impact on the quality of the embryo [33].

In our study, we determined oxidative stress parameters before the beginning of ovarian stimulation and on the day when HCG was administered. We had shown that stimulation in IVF cycles provoked oxidative stress. In both groups there was statistically significant difference in the level of activity of SOD, SH groups and MDA before and after ovarian stimulation, which is consistence with other studies [20] [34]. In one study it has been shown that after stimulationconcentrations of antioxidants were decreased compared to the group with natural cycle [20]. Therefore, this decrease of antioxidants after stimulation may be due to the administration of GT, which is constant with the results that our study gained.

Other authors showed different results when examining the connection between OS and ovarian response to stimulation by gonadotropins in patients in IVF cycles [26]. On oocyte retrieval day samples from serum and follicular fluid were collected. There was a good connection between the level of total antioxidative capacity in plasma and FF which indicated that the system OS may affect the oxidative status in the follicular fluid. This study showed that ovarian stimulation causes an increase in the activity of antioxidants.

There was a larger number of patients who were smokers in group II than in group I (29.4% vs 25.4), but was no significant difference. A larger patient sample might show different results. Different lifestyle habits such as smoking cigarettes are as well associated with oxidative disorders and could have a detrimental effect on reproductive outcomes. Cigarette smoke contains different toxins and free radicals, acting on the oxygenation of cells and tissues. A meta-analysis that included 21 studies published significantly reduced chances of live birth and pregnancy rate per cycle in smokers rather than in non-smokers, as well as a higher rate of miscarriages and pregnancies that were ectopic. In other ART studies, a lower fertilization rate was seen in smokers [35]. Tiboni et al. [35] found in follicular fluid increased levels of lipid peroxidation and lower local antioxidant capacity, due to smoke exposure.

Concerning basal hormonal status, there was no difference between the value of E2 and FSH, butthere was a statistically significant difference in the values of AMH (p = 0.021). AMH values were significantly higher in group I, which was expected for high responders, so a lower dose of gonadotropin for stimulation was needed. Patients in groups I and II had no significantly different number of fertilized oocytes, fertilization rate, number and quality of oocytes. The reason is also that good ovarian response needed a lower dose of therapy. In the two groups no difference was found in the OS parameters, which suggests that the dose of gonadotropin does not impair oxidantantioxidant balance.

Still, ovarian stimulation changes the oxidative status, meaning that IVF protocols should be revised in order to lower excess production of ROS.

When we divided the patients into those who had high parameters of oxidative stress and those who did not before starting the stimulation, we obtained an interesting finding that patients without oxidative stress before entering the IVF procedure needed lower doses of gonadotropins during stimulation.

However, in the study of Velhut et al. importance of intrafollicular and systemic OS and antioxidant response to ovarian stimulation and outcome of ICSI procedure were determined [27] [36]. They concluded that oxidative stress is favorable for the outcome of ovarian stimulation, while for achieving pregnancy a greater overall antioxidant status in plasma was beneficial. Jozwick et al. [13] found no link between OS markers and pregnancy outcomes, but Di Rosa et al. [37] confirmed a highly positive statistically significant connection between the level of OS markers in the serum and the outcome of pregnancies. We should also consider that many other variables can affect the outcome of pregnancies in the IVF program.

To sum up, our results indicate that there is a change in oxidative and antioxidative parameters in serum in the beginning of the cycle and at the end of ovarian stimulation in both lower and higher recombinant FSH dose groups. However, a dose of recombinant FSH is not correlated with a change of the parameters of oxidative stress and antioxidant response in serum, neither with oocytes and embryo quality nor with fertilization, pregnancies, and miscarriages rate. Patients without oxidative stress before entering the IVF procedure needed lower doses of gonadotropins during stimulation.

## Dodatak

### Financial support and sponsorship

None.

### Conflict of interest statement

All the authors declare that they have no conflict of interest in this work.
